# Maternal dietary patterns during pregnancy and preterm delivery: a large prospective cohort study in China

**DOI:** 10.1186/s12937-018-0377-3

**Published:** 2018-07-25

**Authors:** Min-Shan Lu, Jian-Rong He, Qiaozhu Chen, Jinhua Lu, Xueling Wei, Qianling Zhou, Fanfan Chan, Lifang Zhang, Niannian Chen, Lan Qiu, Mingyang Yuan, Kar Keung Cheng, Huimin Xia, Xiu Qiu, on behalf of the Born in Guangzhou Cohort Study Group, Yashu Kuang, Bing Huang, Shenghui Li, Xiaoyan Xia, Yanyan Wu, Songying Shen, Wanqing Xiao, Huiyun Xiao, Huihui Liu, Fengjuan Zhou, Si Tu, Dongmei Wei, Zhaohuan Gui, Yanfei Xing, Lisha Zhu, Xian Liu, Yan Hu, Ying Ma, Weidong Li, Yan Li, Xingwen Zou, Lin Jiang, Jing Zhao, Yi Hu

**Affiliations:** 1 Division of Birth Cohort Study, Guangzhou Women and Children’s Medical Center, Guangzhou Medical University, 9 Junsui Road, Zhujiang Newtown, Tianhe District, Guangzhou, 510623 China; 2 Department of Woman and Child Health Care, Guangzhou Women and Children’s Medical Center, Guangzhou Medical University, Guangzhou, China; 3 Department of Obstetrics and Gynecology, Guangzhou Women and Children’s Medical Center, Guangzhou Medical University, Guangzhou, China; 4 Institute of Applied Health Research, University of Birmingham, Birmingham, UK; 5 Department of Neonatal Surgery, Division of Birth Cohort Study, Guangzhou Women and Children’s Medical Center, Guangzhou Medical University, 9 Junsui Road, Zhujiang Newtown, Tianhe District, Guangzhou, 510623 China

**Keywords:** Pregnant women, Dietary pattern, Preterm delivery, Cluster analysis, Birth cohort, Chinese women

## Abstract

**Background:**

Evidence about the associations between maternal dietary patterns and preterm delivery is scarce in Eastern countries. The purpose of this study was to examine the associations between maternal dietary patterns during pregnancy and preterm delivery in a Chinese population.

**Methods:**

A total of 7352 mothers were included in the Born in Guangzhou Cohort Study, a prospective study in China. A validated self-administered food frequency questionnaire (FFQ) was used to assess maternal diet at 24–27 weeks of gestation. Dietary patterns were identified by cluster analysis. Gestational age was obtained from routine medical records. Preterm delivery was defined as delivery before 37 completed weeks of gestation, and was further classified into spontaneous and iatrogenic preterm delivery, and also early/moderate and late preterm delivery. Associations between dietary patterns and preterm delivery outcomes were assessed using logistic regression analyses.

**Results:**

Six dietary patterns were identified, including ‘Milk’, ‘Cereals, eggs, and Cantonese soups’, ‘Meats’, ‘Fruits, nuts, and Cantonese desserts’, ‘Vegetables’, and ‘Varied’. There were 351 (4.8%) preterm deliveries in this study population. Among those of preterm delivery, 16.2 and 83.8% were early/moderate and late preterm delivery, respectively. Compared with women of ‘Vegetables’ pattern, those of ‘Milk’ pattern had greater odds of overall preterm delivery (adjusted odds ratio [OR] 1.59, 95% confidence interval [CI] 1.11, 2.29, *p* < 0.05), spontaneous preterm delivery (adjusted OR 1.73, 95% CI 1.14, 2.62, *p* < 0.05) and late preterm delivery (adjusted OR 1.73, 95% CI 1.08, 2.62, *p* < 0.05); those of ‘Cereals, eggs, and Cantonese soups’ and ‘Fruits, nuts, and Cantonese desserts’ patterns had greater odds of late preterm delivery (adjusted OR 1.54, 95% CI 1.01, 2.35 for ‘Cereals, eggs, and Cantonese soups’, adjusted OR 1.61, 95% CI 1.04, 2.50 for ‘Fruits, nuts, and Cantonese desserts’, respectively).

**Conclusion:**

Maternal diet with frequent consumption of milk and less frequent consumption of vegetables during pregnancy might be associated with increased odds of preterm delivery. Future interventions should investigate whether increasing vegetable intake reduces preterm deliveries.

**Electronic supplementary material:**

The online version of this article (10.1186/s12937-018-0377-3) contains supplementary material, which is available to authorized users.

## Background

Preterm delivery, defined as birth before 37 completed weeks of gestation, is associated with short-term and long-term neonatal morbidity, and is one of the leading causes of neonatal mortality [1, 2]. Women who deliver preterm have a higher risk of developing cardiovascular disease than those who deliver at full-term [3]. Maternal nutrition can directly affect the growing fetus [3, 4] and considerable amount of evidence has strongly supported the role of diet in preterm delivery [5, 6].

The incidence of preterm delivery is about 11.1% globally [2]. There are geographical variations of the prevalence of preterm delivery; and a maximum 10% of preterm births survive in low-resource settings compared with over 90% in high resource countries. China is one of the ten countries with the highest numbers of preterm deliveries [2]. The rate of preterm delivery in China was 7.1% in 2011 [7], and was estimated to increase in future years [8]. Country specific actions might be considered to tackle factors influencing preterm delivery.

As an important modifiable factor, maternal diet has received considerable amount of attention in previous studies of preterm delivery. However, these studies have mainly focused on assessing the associations between single foods or nutrients and preterm delivery, and yielded mixed results [9–11]. It is not yet known whether the associations between maternal nutrition and preterm delivery are due to overall nutrition or deficiency of a particular nutrient [11]. It is challenging to distinguish the specific effects of single foods or nutrients because of their highly interconnected nature [12]. It might therefore be more useful to assess the whole foods or dietary pattern [12], in order to obtain information valuable for nutrition interventions during pregnancy.

Dietary patterns can represent the combined effects of all foods consumed in a person’s diet. To date, few studies investigated the associations between maternal dietary patterns and preterm delivery, and had varied findings [13–17]. Most of the evidence focused in western countries, including Denmark [13], Norway [14], America [15], and Australia [16]; while only one study was conducted among the Asian population [17]. Chia et al. in the Growing Up in Singapore Towards healthy Outcomes study (GUSTO) reported that vegetables, fruits, and white rice consumption is associated with a lower incidence of preterm delivery among a multiethnic sample in Singapore [17]. Dietary habits are population specific. Distinctive differences exist between the Chinese and the Western diets [18]. Chinese pregnant women have complex and diverse eating behaviors, and follow a set of dietary customs which are not extensively explored in the literature [19, 20]. The aim of this study is therefore to examine the associations between dietary patterns during pregnancy among the Chinese pregnant women and the incidence of preterm delivery.

## Methods

### Study design and population

The present study used data from the Born in Guangzhou Cohort Study (BIGCS), an ongoing prospective cohort study conducted in the Guangzhou Women and Children’s Medical Center (GWCMC). The BIGCS aims to elicit the role of social, biological and environmental influences on pregnancy and child health and development. Methods of the BIGCS are detailed elsewhere [21]. Briefly, women were recruited during their first routine antenatal examinations (normally around 16 weeks, Q1) at two campuses of the GWCMC, and followed up at the second trimester (about 24 to 27 weeks, Q2) and at delivery. The inclusion criteria were women of less than 20 weeks gestation, of Chinese nationality, living in Guangzhou, intended to stay in Guangzhou with their child for at least three years after delivery. The protocols of the BIGCS were reviewed and approved in accordance with the standards of the Institutional Ethics Committee of the GWCMC. All participants gave written consent at the time of recruitment.

**Fig. 1:**
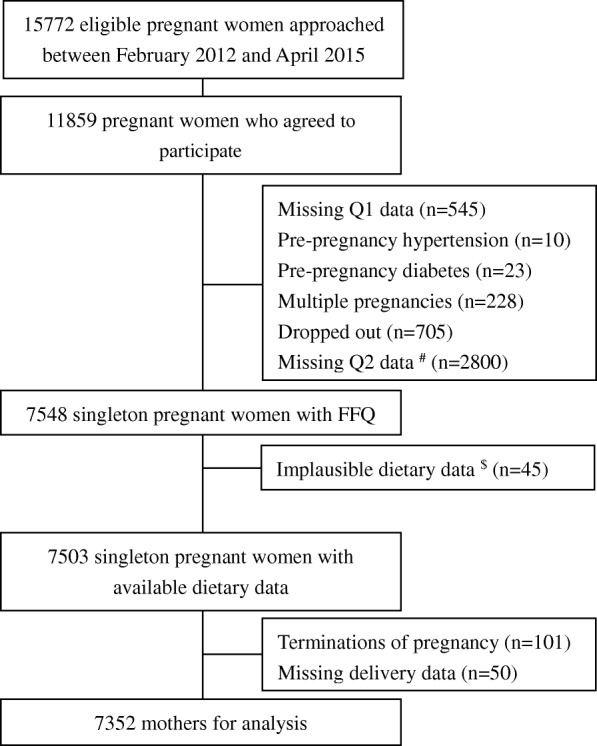
Selection process of study population in the BIGCS. ^#^ There are 2800 participants who did not attend our study clinic in their 24–27 gestational weeks and did not finish the Q2 questionnaire. However, these participants remained in the BIGCS cohort. We excluded them in the current analyses, as dietary information collected in Q2 was the key variable. ^$^ A weekly intake frequency of less than 32 was considered as the ‘implausible dietary intake’ data

A flowchart of the selection process of the study population is shown in Fig. 1. During February 2012 and April 2015, 15,772 eligible pregnant women were invited and 11,859 (75.2%) of them agreed to participate in the BIGCS. After excluding participants who had missing Q1 data (*n* = 545), pre-pregnancy hypertension (*n* = 10), pre-pregnancy diabetes (*n* = 23), occurrence of twin pregnancy (*n* = 228) or dropped out (*n* = 705), missing Q2 data (*n* = 2800), implausible dietary data (a weekly intake frequency of < 32, *n* = 45), terminations of pregnancy (*n* = 101), and missing delivery data (*n* = 50), there were 7352 mothers included in this study. Compared to the remaining 7352 women in the present analysis, women who missed Q2 data were younger, and more likely to have lower levels of education, income and pre-pregnancy body mass index (BMI) (Additional file 1: Table S1).

### Dietary assessment

Information about dietary intake was obtained from a self-administered, non-quantitative food frequency questionnaire (FFQ) at the Q2 interview. This FFQ is a structured questionnaire on 64 specified food items (Additional file 1: Table S2), as well as additional questions regarding cooking oil, beverages, Chinese soup (normally cooked with meat and/or vegetables, of salty flavor and water-like texture [22]), processed meats (such as Lap-mei, Siu mei) and Cantonese desserts. For each food item, participants were asked to indicate their frequency of consumption ‘in the past week’. The FFQ has previously been validated using the BIGCS cohort data [23]. Briefly, a subsample of cohort participants (*n* = 210) completed (1) the first FFQ (FFQ1) at 24–27 weeks of gestation, (2) three inconsecutive 24 h dietary recalls during 29–31 weeks of gestation, and (3) the second FFQ (FFQ2) at 33–35 weeks of gestation. The crude Spearman correlation coefficients for consumption frequencies of food groups between FFQ1 and FFQ2 (0.33–0.71) and between FFQ2 and the average of three 24 h dietary recalls (0.23–0.62) are considered acceptable for dietary assessment during pregnancy [23].

Individual food items were combined into 30 food groups according to a similar nutrient profile or culinary uses (Additional file 1: Table S3). The frequencies of intake of food groups were calculated by summing the weekly consumption frequencies of each food item in the group. The percentages (%) of weekly intake of the food groups were calculated as frequency of the food group intake divided by total frequencies of food intake for each participant. The variables of ‘frequency’ and ‘percentage’ reflect dietary intake from different perspectives. The variable ‘frequency’ reflects the exact frequency of individual food intake; while the variable ‘percentage’ considers the balance of frequencies from different food intakes. Both ‘percentage’ and ‘frequency’ variables were used to construct dietary pattern, respectively, with the application of cluster analysis. Since our purpose was to provide dietary advice for the general pregnant women, we believed the balancing of diet may be more informative than the specific frequency as the ideal amount of food intake should depend on individual energy expenditure status. Therefore, in this paper, results obtained from the ‘percentage’ variables were presented as the main results; while results obtained from the ‘frequency’ variables were presented as supplementary.

Cluster analysis was performed using the k-means procedure, as described elsewhere [24]. The K-means method was applied to classify participants into a predetermined number of mutually exclusive groups, by comparing Euclidean distances between each participant and each cluster center in an interactive process until no further changes occur. Several runs were conducted by varying the number of clusters from two to six, in order to identify the optimal one. The ratios of between-cluster variance to within-cluster variance for each food group [25] were compared across the number of clusters when the variables ‘percentage’ were used as input variables in cluster analysis (Additional file 1: Table S4). We believed that clusters identified from six-cluster solution could better reflect the diversity of participants’ dietary characteristics and are more nutritional meaningful than that from three-cluster solutions. Based on the aforementioned determinations, we selected the six-cluster solution as the most appropriate solution when used ‘percentage’ variables as input variables. We also selected the six-cluster solution when used ‘frequency’ variables as input variables based on the nutritional meaningfulness of clusters. Cluster names were based on the food groups with highest content. These clusters are explained in the result section.

### Assessment of preterm delivery

The primary outcome of this study was preterm delivery, defined as delivery at 36^+ 6^ weeks or below. Gestational age was confirmed by ultrasound examination in the first- or early second-trimester, and was documented in the routine medical record. Secondary outcomes of this study included spontaneous, iatrogenic, early/moderate, and late preterm delivery. Preterm delivery was classified into spontaneous (spontaneous onset of preterm delivery) and iatrogenic (induced or caesarean delivery on maternal or fetal indications) preterm delivery. Preterm delivery was also categorized into early/moderate (≤ 33^+ 6^ weeks) and late (34^+ 0^ to 36^+ 6^ weeks) preterm delivery, according to the gestational age.

### Covariates

Information regarding potential confounders was assessed by self-reported comprehensive baseline questionnaire at Q1, including maternal age, education level (high school or below, vocational/technical college, undergraduate, postgraduate), monthly income (≤1500, 1501–4500, 4501–9000, or ≥ 9001 yuan), parity (primiparous, multiparous), passive smoking during pregnancy (no, yes), supplementation with folic acid (no, started during pregnancy, or started pre- conception), pre-pregnancy weight (kg) and height (m), previous preterm delivery (no, yes). Pre-pregnancy BMI (kg/m^2^) was calculated by dividing weight in kilograms by height in meters squared. Study participants were divided into three groups as follows: BMI < 18.5 kg/m^2^ (underweight), BMI 18.5–23.9 kg/m^2^ (normal), BMI ≥ 24 kg/m^2^ (overweight or obese), according to the Guidelines for Prevention and Control of Overweight and Obesity in Chinese Adults [26].

### Statistical analyses

Descriptive statistics (i.e., mean, standard deviation, frequencies, and percent frequencies) were reported for all socio-demographic factors (age, education level, and monthly income), health characteristics (parity, passive smoking during pregnancy, pre-pregnancy BMI, previous preterm delivery, and supplementation with folic acid). These variables were cross- tabulated by dietary patterns, and significant differences were assessed by ANOVA for continuous variables or chi-square tests for categorical variables. Logistic regression was conducted to assess the independent effect of dietary patterns on preterm delivery related outcomes, including overall preterm delivery, spontaneous preterm delivery, iatrogenic preterm delivery, early/moderate preterm delivery as well as late preterm delivery. All covariates described above were entered into each regression model as potential confounders. The firth’s correction was applied to improve the accuracy of the logit coefficients in the adjusted models referring to iatrogenic preterm delivery and early/moderate preterm delivery.

*P* < 0.05 was considered statistically significant. Cluster analysis was performed by R version 3.2.3 [27], and the remaining analyses were performed using SPSS software version 20.0 (SPSS, Inc., Chicago, USA).

## Results

Six clusters of dietary pattern were identified (Table 1), namely the ‘Milk’ (n 1090, 14.8%), ‘Cereals, eggs, and Cantonese soups’ (n 1078, 14.7%), ‘Meats’ (n 1125, 15.3%), ‘Fruits, nuts, and Cantonese desserts’ (n 875, 11.9%), ‘Vegetables’ (n 1442, 19.6%), and ‘Varied’ (n 1742, 23.7%) patterns. ‘Milk’ had the most frequently consumed of milk products (including fresh milk, pasteurized milk, milk powder, and formula for pregnant women) and less frequently consumed of whole vegetables. ‘Cereals, eggs, and Cantonese soups’ had the most frequently consumed of staples such as rice, pasta, porridge, eggs, and Cantonese soups. ‘Meats’ had the most frequently consumed of red meat and processed meat. ‘Fruits, nuts, and Cantonese desserts’ had the most frequently consumed of fruits, nuts, and Cantonese desserts. ‘Vegetables’ had the most frequently consumed of leafy and cruciferous vegetables. ‘Varied’ was characterized by relatively frequent consumption of mixed foods, including noodles, bread, root vegetables, melon vegetables, mushrooms, sea vegetables, bean vegetables, processed vegetables, poultry, animal organ meat, fish, other seafood, bean products, yoghurt, sweet beverages, puffed food, confectioneries, and snacks.

**Table 1:** Percentages (%) of weekly intake of 30 food groups assessed with a self-administered food frequency questionnaire across the six dietary patterns identified among 7352 pregnant Chinese women from the Born in Guangzhou Cohort Study

	Dietary patterns											
	Varied		Milk		Pattern ^a^		Meats		Pattern ^b^		Vegetables	
	(*n* = 1742)		(*n* = 1090)		(*n* = 1078)		(*n* = 1125)		(*n* = 875)		(*n* = 1442)	
Food groups ^c^	Mean	SD	Mean	SD	Mean	SD	Mean	SD	Mean	SD	Mean	SD
Cereals												
Rice	8.1	2.8	10.9	3.4	16.4	3.8	12.5	3.2	10.4	3.4	12.0	3.5
Pasta	4.4	2.8	4.1	2.8	5.1	3.6	4.2	2.8	4.3	2.9	4.0	2.5
Noodles	2.8	3.0	1.6	2.0	2.3	2.6	2.0	2.1	2.4	2.6	2.0	2.1
Porridge	1.6	1.5	1.3	1.5	1.8	1.8	1.6	1.7	1.3	1.5	1.4	1.5
Bread	2.1	1.8	1.8	2.1	2.0	2.0	1.6	1.8	1.3	1.7	1.9	1.9
Vegetables												
Leafy and cruciferous vegetables	10.1	3.0	10.7	3.4	7.8	2.9	10.5	3.7	9.3	3.4	17.9	3.8
Root vegetables	3.7	1.9	2.5	1.8	2.8	1.8	2.7	1.8	3.0	1.9	3.1	2.0
Melon vegetables	4.2	2.2	3.3	2.1	3.7	2.3	3.5	2.2	4.0	2.4	3.5	2.2
Mushrooms	1.5	1.2	1.0	1.1	1.1	1.1	1.0	1.0	1.2	1.1	1.1	1.1
Sea vegetables	1.0	1.0	0.6	0.8	0.7	0.9	0.6	0.9	0.7	0.9	0.6	0.9
Bean vegetables	1.6	1.2	1.2	1.2	1.3	1.3	1.3	1.2	1.3	1.1	1.3	1.1
Processed vegetables	0.6	1.0	0.3	0.7	0.5	0.9	0.4	0.8	0.4	0.9	0.4	0.8
Fruits	7.7	2.4	8.1	2.9	8.1	2.8	7.5	2.9	15.3	3.9	7.6	2.9
Meats												
Red meat	7.1	2.5	7.7	3.0	6.8	2.7	14.4	3.4	7.1	3.1	7.6	3.0
Poultry	2.6	1.8	2.4	1.7	2.5	1.8	2.5	2.0	2.0	1.6	2.4	1.8
Animal organ meat	1.2	1.5	1.0	1.4	1.0	1.3	1.0	1.4	0.8	1.1	0.9	1.2
Processed meat	0.3	0.7	0.3	0.9	0.3	0.8	0.4	0.9	0.3	0.7	0.2	0.7
Eggs	5.3	2.2	5.8	2.5	5.8	2.9	5.5	2.8	5.3	2.5	5.0	2.3
Fish	3.1	1.9	3.0	2.1	3.0	2.0	2.9	2.0	2.7	1.8	2.9	1.8
Other seafood	1.2	1.3	0.9	1.1	1.0	1.2	0.9	1.1	1.0	1.2	0.9	1.2
Bean products	6.9	3.6	3.4	2.4	4.2	2.8	3.7	2.5	4.4	2.8	3.8	2.5
Nuts	4.3	2.4	4.1	2.6	3.8	2.7	3.3	2.5	4.6	3.1	3.5	2.5
Milk	5.1	2.7	13.0	4.1	5.9	3.0	5.4	2.9	5.9	3.2	5.1	2.9
Yoghurt	2.5	2.2	1.5	1.9	2.0	2.3	1.7	2.0	2.2	2.3	1.8	2.1
Sweet beverages	1.9	3.1	1.2	2.1	1.6	2.6	1.2	1.9	1.4	2.2	1.2	2.0
Cantonese desserts	0.3	0.9	0.3	1.0	0.2	0.7	0.2	0.8	0.4	1.1	0.2	0.7
Cantonese soups	3.1	2.1	3.6	2.3	4.0	2.5	3.7	2.3	3.1	2.3	3.2	2.1
Puffed food	0.3	0.7	0.2	0.5	0.2	0.6	0.2	0.5	0.2	0.5	0.2	0.5
Confectioneries	2.2	2.5	1.3	2.0	1.6	2.2	1.3	1.8	1.7	2.2	1.4	2.0
Snack	3.2	2.4	2.9	2.7	2.9	2.7	2.4	2.3	2.2	2.3	2.7	2.3

^a^ “Cereals, eggs and Cantonese soups”

^b^ “Fruits, nuts and Cantonese desserts”

^c^ Percentage values (%), calculated as frequency of the food group intake divided by total frequencies of food intake. The highest mean values are underlined

Subject characteristics across the six dietary patterns are shown in Table 2. There were significant differences in maternal age, education level, monthly income, parity, and passive smoking during pregnancy among subjects in these six groups. No significant difference regarding supplementation with folic acid, pre-pregnancy BMI and previous preterm delivery was found among subjects in these six groups.

**Table 2:** Characteristics of the participants across the six dietary patterns identified by cluster analysis

		Dietary patterns						*P*_value_*
	Total	Varied	Milk	Cereals, eggs and Cantonese soups	Meats	Fruits, nuts and Cantonese desserts	Vegetables	
Characteristics	(*n* = 7352)	(*n* = 1742)	(*n* = 1090)	(*n* = 1078)	(*n* = 1125)	(*n* = 875)	(*n* = 1442)	
Age, years, mean ± SD	29.1 ± 3.3	29.3 ± 3.3	29.0 ± 3.5	28.8 ± 3.3	29.0 ± 3.3	29.0 ± 3.2	29.3 ± 3.4	0.002
Education level, n (%)								< 0.001
High school or below	624 (8.5)	117 (6.7)	105 (9.6)	110 (10.2)	83 (7.4)	79 (9.0)	130 (9.0)	
Vocational/technical college	1807 (24.6)	338 (19.4)	285 (26.1)	309 (28.7)	282 (25.1)	213 (24.3)	380 (26.4)	
Undergraduate	4031 (54.8)	986 (56.6)	591 (54.2)	569 (52.8)	642 (57.1)	461 (52.7)	782 (54.2)	
Postgraduate	890 (12.1)	301 (17.3)	109 (10.0)	90 (8.3)	118 (10.5)	122 (13.9)	150 (10.4)	
Monthly income, Yuan, n (%)								< 0.001
≤ 1500	692 (9.4)	161 (9.2)	107 (9.8)	93 (8.6)	101 (9.0)	92 (10.5)	138 (9.6)	
1501–4500	2274 (30.9)	430 (24.7)	402 (36.9)	387 (35.9)	383 (34.0)	216 (24.7)	456 (31.6)	
4501–9000	3062 (41.6)	754 (43.3)	410 (37.6)	441 (40.9)	453 (40.3)	396 (45.3)	608 (42.2)	
≥ 9001	1158 (15.8)	353 (20.3)	143 (13.1)	140 (13.0)	164 (14.6)	150 (17.1)	208 (14.4)	
Refused to answer	166 (2.3)	44 (2.5)	28 (2.6)	17 (1.6)	24 (2.1)	21 (2.4)	32 (2.2)	
Parity, n (%)								< 0.001
Primiparous	6430 (87.5)	1501 (86.2)	1002 (91.9)	967 (89.7)	956 (85.0)	799 (91.3)	1205 (83.6)	
Multiparous	922 (12.5)	241 (13.8)	88 (8.1)	111 (10.3)	169 (15.0)	76 (8.7)	237 (16.4)	
Passive smoking during pregnancy, n (%)	2222 (30.2)	472 (27.2)	322 (29.5)	364 (33.8)	384 (34.1)	252 (28.8)	428 (29.7)	< 0.001
Supplementation with folic acid, n (%)								0.110
No	603 (8.2)	128 (7.3)	91 (8.3)	96 (8.9)	103 (9.2)	66 (7.5)	119 (8.3)	
Started during pregnancy	3501 (47.6)	811 (46.6)	511 (46.9)	535 (49.6)	535 (47.6)	392 (44.8)	717 (49.7)	
Started pre- conception	3248 (44.2)	803 (46.1)	488 (44.8)	447 (41.5)	487 (43.3)	417 (47.7)	606 (42.0)	
Pre-pregnancy BMI, kg/m^2^, n (%)								0.325
< 18.5	1803 (24.5)	414 (23.8)	293 (26.9)	274 (25.4)	257 (22.8)	212 (24.2)	353 (24.5)	
18.5–23.9	4608 (62.7)	1114 (63.9)	645 (59.2)	682 (63.3)	705 (62.7)	563 (64.3)	899 (62.3)	
≥ 24	847 (11.5)	189 (10.8)	137 (12.6)	110 (10.2)	145 (12.9)	93 (10.6)	173 (12.0)	
Missing	94 (1.3)	25 (1.4)	15 (1.4)	12 (1.1)	18 (1.6)	7 (0.8)	17 (1.2)	
Previous preterm delivery, n (%)	53 (0.7)	14 (0.8)	9 (0.8)	5 (0.5)	8 (0.7)	3 (0.3)	14 (1.0)	0.502

*ANOVA and Chi square tests were used to test differences between the patterns

The gestational length range was 27^+ 6^ to 42^+ 1^ weeks in our study. There were 351 women delivered preterm, taking up 4.8% of the total participants. Among these 351 women, 262 (74.6%) were spontaneous preterm delivery, 62 (17.7%) were iatrogenic preterm delivery, and the rest 27 (7.7%) cases were either spontaneous or iatrogenic preterm delivery. We are unable to classify these 27 cases because they did not deliver their babies at the GWCMC. Among 351 women who delivered preterm, 294 (83.8%) were late preterm and 57 (16.2%) were moderately or early preterm. The incidence of preterm delivery was highest (66 cases, 6.1%) in the ‘Milk’ group. Table 3 shows associations between dietary patterns and preterm delivery. Because the beneficial value of vegetables, we selected ‘Vegetables’ pattern as reference pattern. Compared with women in the ‘Vegetables’ group (reference), those in the ‘Milk’ group had significantly higher odds of preterm delivery after adjustment for potential confounders (OR 1.59, 95% CI 1.11, 2.29, *p* < 0.05). No significant difference in the odds of preterm delivery was observed among subjects in other patterns neither in the crude nor adjusted models.

**Table 3:** Associations between dietary patterns and preterm delivery

Preterm delivery	Dietary patterns					
	Varied	Milk	Cereals, eggs and Cantonese soups	Meats	Fruits, nuts and Cantonese desserts	Vegetables
Overall preterm delivery (n, %)	85 (4.9)	66 (6.1)	54 (5.0)	44 (3.9)	43 (4.9)	59 (4.1)
Crude OR (95% CI)	1.20 (0.86–1.69)	1.51 (1.05–2.17) ^*^	1.24 (0.85–1.80)	0.95 (0.64–1.42)	1.21 (0.81–1.81)	1.00 (Reference)
Adjusted OR (95% CI) ^a^	1.27 (0.90–1.80)	1.59 (1.11–2.29) ^*^	1.31 (0.89–1.92)	1.01 (0.67–1.51)	1.30 (0.87–1.96)	1.00 (Reference)
Spontaneous preterm delivery (n, %)	63 (3.7)	52 (4.8)	39 (3.7)	32 (2.9)	34 (3.9)	42 (2.9)
Crude OR (95% CI)	1.25 (0.84–1.86)	1.67 (1.10–2.53) ^*^	1.25 (0.81–1.95)	0.97 (0.61–1.55)	1.35 (0.85–2.13)	1.00 (Reference)
Adjusted OR (95% CI) ^a^	1.29 (0.86–1.92)	1.73 (1.14–2.62) ^*^	1.30 (0.83–2.03)	1.00 (0.63–1.60)	1.41 (0.89–2.24)	1.00 (Reference)
Iatrogenic preterm delivery (n, %)	14 (0.8)	9 (0.9)	10 (1.0)	5 (0.5)	8 (1.0)	16 (1.1)
Crude OR (95% CI)	0.73 (0.36–1.50)	0.76 (0.33–1.73)	0.84 (0.38–1.87)	0.40 (0.15–1.09)	0.83 (0.35–1.95)	1.00 (Reference)
Adjusted OR (95% CI) ^a, b^	0.79 (0.38–1.61)	0.79 (0.34–1.74)	0.90 (0.40–1.94)	0.46 (0.16–1.14)	0.87 (0.36–1.97)	1.00 (Reference)
Late preterm delivery (n, %)	73 (4.2)	53 (4.9)	47 (4.4)	37 (3.3)	40 (4.6)	44 (3.1)
Crude OR (95% CI)	1.38 (0.95–2.03)	1.63 (1.08–2.45) ^*^	1.44 (0.95–2.19)	1.08 (0.69–1.68)	1.51 (0.98–2.34)	1.00 (Reference)
Adjusted OR (95% CI) ^a^	1.46 (0.99–2.15)	1.73 (1.08–2.62) ^*^	1.54 (1.01–2.35) ^*^	1.11 (0.73–1.79)	1.61 (1.04–2.50) ^*^	1.00 (Reference)
Moderately or early preterm delivery (n, %)	12 (0.7)	13 (1.3)	7 (0.7)	7 (0.6)	3 (0.4)	15 (1.1)
Crude OR (95% CI)	0.67 (0.31–1.43)	1.17 (0.55–2.47)	0.63 (0.26–1.55)	0.60 (0.24–1.47)	0.33 (0.10–1.15)	1.00 (Reference)
Adjusted OR (95% CI) ^a, b^	0.73 (0.34–1.55)	1.19 (0.56–2.50)	0.68 (0.27–1.58)	0.63 (0.25–1.48)	0.42 (0.11–1.21)	1.00 (Reference)

^a^ Adjusted for maternal age, education level, monthly income, parity, passive smoking during pregnancy, supplementation with folic acid, pre-pregnancy BMI, and previous preterm delivery

^b^ The firth’s correction was applied to improve the accuracy of the logit coefficients

^*^ *P*_value_ < 0.05

When we analyzed secondary outcomes individually, we found significantly greater odds of spontaneous preterm delivery for women in the ‘Milk’ groups in comparison to those in the ‘Vegetables’ groups (adjusted OR 1.73, 95% CI 1.14, 2.62, *p* < 0.05). No significant association was found between iatrogenic preterm delivery and maternal dietary pattern. We also found significantly greater odds of late preterm delivery for women in the ‘Milk’ (adjusted OR 1.73, 95% CI 1.08, 2.62, *p* < 0.05), ‘Cereals, eggs, and Cantonese soups’ (adjusted OR 1.54, 95% CI 1.01, 2.35, *p* < 0.05) and ‘Fruits, nuts, and Cantonese desserts’ (adjusted OR 1.61, 95% CI 1.04, 2.50, *p* < 0.05) groups in comparison to those in the ‘Vegetables’ groups. No significant association was found between moderately or early preterm delivery and maternal dietary pattern (Table 3).

By using ‘frequencies’ of 30 food groups as input variables in the cluster analysis, we also identified six dietary patterns and labeled them as ‘Rich’ (n 381, 5.2%), ‘Milk-S’ (n 864, 11.8%), ‘Fruits’ (n 930, 12.6%), ‘Meats-S’ (n 975, 13.3%), ‘Moderate’ (n 1735, 23.6%), and ‘Prudent’ (n 2467, 33.6%) patterns (Additional file 1: Table S5). The letter “S” was added after the pattern name (e.g. “Milk-S” and “Meats-S”) to separate supplementary (from ‘frequency’ variables) and main (from ‘percentage’ variables) results. Additional file 1: Table S6 presents subject characteristics across the six dietary patterns. No significant association was found between these dietary patterns and overall preterm delivery. Compared with women in other dietary patterns, women in the ‘Milk-S’ group had significantly higher odds of spontaneous preterm delivery (adjusted OR 1.44, 95% CI 1.02, 2.02, *p* < 0.05), while women in the ‘Rich’ group had significantly lower odds of spontaneous preterm delivery (adjusted OR 0.41, 95% CI 0.18, 0.93, *p* < 0.05) (Additional file 1: Table S7).

## Discussion

This is the first prospective study to examine the relationships between maternal dietary patterns and preterm delivery in a Chinese population with a relatively large sample size. Six dietary patterns of the Chinese were generated, represented by foods generally consumed by the Chinese frequently and the cultural Cantonese cuisine. Women in the ‘Milk’ group had greater odds of overall preterm delivery, spontaneous preterm delivery and late preterm delivery than those in the ‘Vegetables’ group. We also found that, compared with women in the ‘Vegetables’ group, those in the ‘Cereals, eggs, and Cantonese soups’ and ‘Fruits, nuts, and Cantonese desserts’ groups had increased odds of late preterm delivery.

Our findings implied that maternal diet with frequent consumption of vegetables might contribute to lower odds of preterm delivery. Similarly, several studies suggested that vegetables are important components of protective dietary patterns to which women adhere may have lower odds of preterm delivery. In a large prospective cohort study in Norway, diets rich in vegetables and fruits, known as the prudent diet, are associated with a lower incidence of preterm delivery [14]. Another study in Singapore has shown that a dietary pattern high in vegetables, fruits, and white rice is associated with a lower incidence of preterm delivery [17]. Low vegetables intake might induce to inadequacy of antioxidants, which can reduce both systemic and local inflammation [28] and hence the risk of preterm premature rupture of membranes [29]. Insufficient intake of vegetables inhibits peristalsis and might lead to constipation during pregnancy [30, 31], which might further impair fetal growth [32].

Frequent consumption of milk and less frequent consumption of vegetables in our study was found to have higher odds of preterm delivery. Similarly, increased odds of preterm delivery were also found for a diet high in whole milk in the American population [15]. Our findings could be explained from the following aspects. Firstly, dairy is not consumed on a regular or daily basis for most of the Chinese [19]. The variety of dairy products in China is not as much as that in the Western countries. Milk (mainly cow’s milk), including fresh milk and milk powder, is the main source of dairy products in China [18]. Yoghurt consumption is increasing in China; however, there is still a gap to catch up with the Western world. Dairy products (e.g. milk, cheese and yoghurt), high in dietary protein and calcium, are essential for fetal growth and skeletal development [33, 34]. Dairy is thus recommended in pregnancy dietary guidelines in different countries including China [35, 36]. Women in the ‘Milk’ group tended to consume whole milk more frequently, milk powder while they had yoghurt less frequently in their diet (Table 1). Yoghurt products enriched with probiotics have been reported to be associated with a reduced risk of preterm delivery [9, 37]. Another possible explanation is that women having a frequent consumption of milk in our study might thus consider their diet healthy enough, without paying attention to the context of a balanced diet and the whole vegetables consumption.

In the subgroup analyses, we only found a significant association between dietary pattern and spontaneous preterm delivery for women in the ‘Milk’ groups in comparison to those in the ‘Vegetables’ groups. Our supplementary results obtained from ‘frequency’ variable support such finding. After stratification of preterm delivery according to gestational age, we only found significantly greater odds of late preterm delivery for women in the ‘Milk’, ‘Cereals, eggs, and Cantonese soups’ and ‘Fruits, nuts, and Cantonese desserts’ groups in comparison to those in the ‘Vegetables’ groups. In agreement with our study, several studies also showed that the significant association was primarily driven by the higher incidence of spontaneous preterm delivery [14, 15, 17] and late preterm delivery [14]. It is speculated that dietary factors might only marginally reduce the progression to preterm delivery and the effect is therefore most easily detectable in late preterm delivery [14]. It appears that there is no significant association between maternal diet and iatrogenic preterm delivery or early/moderately preterm delivery in our study. Notably, only a single measure of dietary intake obtained from an FFQ was used in this study, limiting the validity of the finding.

This is the first prospective study to examine the relationship between maternal dietary patterns and preterm delivery in a Chinese population with a relatively large sample size. The high participation rate in the cohort study and the availability of ultrasound data to confirm gestational age are additional major strengths. Previous studies exploring maternal dietary patterns and preterm delivery have mostly been conducted by using factor analysis to identify dietary patterns. Instead, we used cluster analysis, which can provide a clear description of exactly what is frequently consumed [38]. Our findings might thus be more valuable for a nutrition intervention design to target pregnant women in need. In addition, patterns obtained from cluster analysis in our study appear to better reflect the Asian dietary pattern than that from factor analysis conducted by He et al. in the same cohort [39].

The present study had some limitations. Firstly, we did not collect data on food servings or portion sizes of food items, and were unable to calculate the amount of food consumption and adjust the total energy intake. However, it was suggested that individuals are generally incapable to describe food portions accurately [40], and there are sustainable within subject variations in the indication of food quantities [41]. In contrast, a simple FFQ is sufficient to indicate actual intakes [42]. As previous studies [39, 43], frequencies of food intake were used as a proxy for a quantitative indicator. Secondly, we assessed food intake ‘in the past week’ at 24–28 week of gestation. The information during this short period might not be representative of dietary habits throughout pregnancy. However, previous studies have suggested that overall dietary patterns differed minimally during pregnancy [44, 45]. Thirdly, we could not precisely distinguish women who were on special diets, which might affect the findings. However, such case is estimated to be rare in our study, as we have excluded women with pre-pregnancy hypertension or diabetes. Only four participants were reported as ‘vegan’. Fourthly, more than 20% of the participants missed Q2 data. Finally, owing to the nature of the observational study design, we are unable to identify the causality between preterm delivery and diet. Residual confounders are likely to exist even after we have adjusted for several factors in the statistical analysis.

## Conclusions

In conclusion, a maternal pregnancy diet with frequent consumption of milk and less frequent consumption of vegetables is found to be associated with increased odds of preterm delivery among Chinese women in the current large-scale birth cohort. Frequent consumption of vegetables should be recommended during pregnancy to prevent preterm delivery.

## Additional file

Additional file 1: **Table S1.** Comparison of characteristics among women remained in the present study and those who missed Q2 data. **Table S2.** Food List in the food frequency questionnaire (FFQ) of BIGCS. **Table S3.** List of food items included in the 30 main food groups. **Table S4** The ratios of between-cluster variance to within-cluster variances for each food group across clusters from two to six. **Table S5.** Frequencies of weekly intake of 30 food groups assessed with a self-administered food frequency questionnaire across the six dietary patterns identified among 7352 pregnant Chinese women from the Born in Guangzhou Cohort Study. **Table S6.** Characteristics of the participants across the six dietary patterns identified by cluster analysis. **Table S7.** Associations between dietary patterns and preterm delivery. (DOCX 54 kb)

## Abbreviations

ANOVA: One-way analysis of variance
BIGCS: Born in Guangzhou cohort study
BMI: Body mass index
CI: Confidence interval
FFQ: Food frequency questionnaire
GWCMC: Guangzhou women and children’s medical center
OR: Odds ratios
SD: Standard deviation
