# Supplementary material for: Maternal dietary patterns during pregnancy and preterm delivery: a large prospective cohort study in China
Source: Nutr J. 2018 Jul 25;17:71. doi: 10.1186/s12937-018-0377-3 (PMC6060524; doi:10.1186/s12937-018-0377-3)
Supplement: Supplementary file 1 — Table S1. Comparison of characteristics among women remained in the present study and those who missed Q2 data. Table S2. Food List in the food frequency questionnaire (FFQ) of BIGCS. Table S3. List of food items included in the 30 main food groups. Table S4 The ratios of between-cluster variance to within-cluster variances for each food group across clusters from two to six. Table S5. Frequencies of weekly intake of 30 food groups assessed with a self-administered food frequency questionnaire across the six dietary patterns identified among 7352 pregnant Chinese women from the Born in Guangzhou Cohort Study. Table S6. Characteristics of the participants across the six dietary patterns identified by cluster analysis. Table S7. Associations between dietary patterns and preterm delivery. (DOCX 54 kb) [file 12937_2018_377_MOESM1_ESM.docx]

**Table S1** Comparison of characteristics among women remained in the present study and those who missed Q2 data

| Characteristics | Remained in the present study  (n=7352) | Missed Q2 data  (n=2800) |  | *P*_value_* |
| --- | --- | --- | --- | --- |
| Age, years, mean ± SD | 29.1 ± 3.3 | 28.5 ± 3.4 |  | <0.001 |
| Education level, n (%) |  |  |  | <0.001 |
| High school or below | 624 (8.5) | 409 (14.6) |  |  |
| Vocational/technical college | 1807 (24.6) | 775 (27.7) |  |  |
| Undergraduate | 4031 (54.8) | 1327 (47.4) |  |  |
| Postgraduate | 890 (12.1) | 289 (10.3) |  |  |
| Monthly income, Yuan, n (%) | | |  | 0.006 |
| ≤1500 | 692 (9.4) | 328 (11.7) |  |  |
| 1501-4500 | 2274 (30.9) | 844 (30.1) |  |  |
| 4501-9000 | 3062 (41.6) | 1103 (39.4) |  |  |
| ≥9001 | 1158 (15.8) | 457 (16.3) |  |  |
| Refused to answer | 166 (2.3) | 68 (2.4) |  |  |
| Pre-pregnancy BMI, kg/m2, n (%) | | |  | 0.005 |
| <18.5 | 1803 (24.5) | 776 (27.7) |  |  |
| 18.5-23.9 | 4608 (62.7) | 1699 (60.7) |  |  |
| ≥24 | 847 (11.5) | 285 (10.2) |  |  |
| Missing | 94 (1.3) | 40 (1.4) |  |  |

*Significant differences across groups were tested by using t test or Chi square tests.

**Table S2 Food List in the food frequency questionnaire (FFQ) of BIGCS**

**1. Staple food**

1.1 Rice

1.2 Pasta

1.3 Porridge

1.4 Rice noodles

1.5 Starchy noodles

1.6 Coarse cereal

1.7 Others

**2. Meat**

2.1 Pork

2.2 Beef

2.3 Mutton

2.4 Poultry

2.5 Others

**3. Organs/blood**

3.1 Animal liver

3.2 Other animal innards: heart/kidney/intestine

3.3 Animal brain

3.4 Animal blood

**4. Egg**

4.1 Fresh egg

4.2 Preserved egg

**5. Fish/****sea food**

5.1 Freshwater fish

5.2 Seawater fish

5.3 Prawn/crab

5.4 Shell fish/squid

5.5 Others

**6. Bean products**

6.1 Soybean

6.2 Other dry beans: mung bean /red bean/brown bean

6.3 Soybean milk

6.4 Bean curd

6.5 Fresh green beans

**7. Dairy**

7.1 Fresh milk

7.2 Yogurt

7.3 Pasteurized milk

7.4 Formula milk powder

7.5 Fat free milk powder

7.6 Whole milk powder

7.7 Others

**8. Vegetables**

8.1 Dark green leafy vegetables

8.2 White leafy vegetables

8.3 Broccoli

8.4 Cauliflower

8.5 Carrots

8.6 Potatoes/Radishes/ lotus root

8.7 Pumpkin/tomato/ capsicums /eggplant

8.8 Wax gourd/ cucumber/ white eggplant/ bitter gourd

8.9 Mushrooms

8.10 Processed vegetables

8.11 Sea vegetables

8.12 Others

**9. Fruits**

9.1 Cherry/ grapefruit/plum/apple/pear/peach

9.2 Banana/oranges/grape/ kiwifruit

9.3 Watermelon/pineapple/mango/litchi/longan/durian

9.4 Others

**10. Nuts**

10.1 Oil nuts: peanut/walnut/almonds/pistachios

10.2 Starchy nuts: chestnuts

**11. Candy/ Dessert /Snack**

11.1 Honey

11.2 Candy/ Chocolate

11.3 Confectioneries

11.4 Puffed food

11.5 Others

**12.** Bread/ Cakes /Fast food

12.1 Bread

12.2 Biscuit

12.3 Cornmeal

12.4 Cake/fried dough twist

12.5 Moon cake/rice dumpling

12.6 Others

**Table S3 List of food items included in the 30 main food groups**

| Food groups | Food items in the FFQ |
| --- | --- |
| **Cereals** |  |
| Rice | 1.1 Rice |
| Pasta | 1.2 Pasta |
| Noodles | 1.3 Porridge |
| Porridge | 1.4 Rice noodles  1.5 Starchy noodles |
| Bread | 12.1 Bread |
| **Vegetables** |  |
| Leafy and cruciferous vegetables | 8.1 Dark green leafy vegetables  8.2 White leafy vegetables  8.3 Broccoli  8.4 Cauliflower |
| Root vegetables | 8.5 Carrots  8.6 Potatoes/Radishes/ lotus root  8.7 Pumpkin/tomato/ capsicums /eggplant |
| Melon vegetables | 8.8 Wax gourd/ cucumber/ white eggplant/ bitter gourd |
| Mushrooms | 8.9 Mushrooms |
| Sea vegetables | 8.11 Sea vegetables |
| Bean vegetables | 6.5 Fresh green beans |
| Processed vegetables | 8.10 Processed vegetables |
| **Fruits** | 9.1 Cherry/ grapefruit/plum/apple/pear/peach  9.2 Banana/oranges/grape/ kiwifruit  9.3 Watermelon/pineapple/mango/litchi/longan/durian  9.4 Others |
| **Meats** |  |
| Red meat | 2.1 Pork  2.2 Beef  2.3 Mutton  2.5 Others |
| Poultry | 2.4 Poultry |
| Animal organ meat | 3.1 Animal liver  3.2 Other animal innards: heart/kidney/intestine  3.3 Animal brain  3.4 Animal blood |
| Processed meat | Additional questions: processed meats (such as Lap-mei, Siu mei) |
| Eggs | 4.1 Fresh egg  4.2 Preserved egg |
| Fish | 5.1 Freshwater fish  5.2 Seawater fish |
| Other seafood | 5.3 Prawn/crab  5.4 Shell fish/squid  5.5 Others |
| **Bean products** | 6.1 Soybean  6.2 Other dry beans: mung bean /red bean/brown bean  6.3 Soybean milk  6.4 Bean curd |
| **Nuts** | 10.1 Oil nuts: peanut/walnut/almonds/pistachios  10.2 Starchy nuts: chestnuts |
| **Milk** | 7.1 Fresh milk  7.3 Pasteurized milk  7.4 Formula milk powder  7.5 Fat free milk powder  7.6 Whole milk powder  7.7 Others |
| Yoghurt | 7.2 Yogurt |
| Sweet beverages | Additional questions: sweet beverages |
| Cantonese desserts | Additional questions: Cantonese desserts |
| Cantonese soups | Additional questions: Cantonese soups |
| Puffed food | Puffed food |
| Confectioneries | 11.1 Honey  11.2 Candy/ Chocolate  11.3 Confectioneries  11.5 Others |
| Snack | 12.2 Biscuit  12.3 Cornmeal  12.4 Cake/fried dough twist  12.5 Moon cake/rice dumpling  12.6 Others |

**Table S4** The ratios of between-cluster variance to within-cluster variances for each food group across clusters from two to six

|  | “Percentage” | | | | |  | “Frequency” | | | | |
| --- | --- | --- | --- | --- | --- | --- | --- | --- | --- | --- | --- |
|  | Cluster Number | | | | |  | Cluster Number | | | | |
| Food groups | 2 | 3 | 4 | 5 | 6 |  | 2 | 3 | 4 | 5 | 6 |
| Rice | 6 | 1165 | 619 | 943 | 872 |  | 303 | 270 | 191 | 147 | 161 |
| Pasta | 48 | 22 | 12 | 11 | 20 |  | 211 | 156 | 109 | 71 | 58 |
| Noodles | 34 | 84 | 79 | 56 | 40 |  | 125 | 70 | 43 | 60 | 61 |
| Porridge | 2 | 2 | 8 | 8 | 16 |  | 82 | 64 | 34 | 30 | 29 |
| Bread | 6 | 5 | 8 | 7 | 26 |  | 156 | 132 | 87 | 65 | 55 |
| Leafy and cruciferous vegetables | 8071 | 3505 | 2188 | 1663 | 1471 |  | 4699 | 4829 | 5246 | 3708 | 2654 |
| Root vegetables | 1 | 109 | 84 | 74 | 72 |  | 1039 | 685 | 418 | 339 | 271 |
| Melon vegetables | 18 | 54 | 51 | 34 | 30 |  | 926 | 646 | 420 | 320 | 260 |
| Mushrooms | 2 | 64 | 46 | 40 | 40 |  | 432 | 293 | 206 | 165 | 142 |
| Sea vegetables | 13 | 57 | 45 | 40 | 33 |  | 249 | 174 | 117 | 96 | 87 |
| Bean vegetables | 6 | 24 | 18 | 19 | 18 |  | 459 | 282 | 165 | 141 | 104 |
| Processed vegetables | 2 | 24 | 27 | 26 | 19 |  | 41 | 30 | 19 | 28 | 26 |
| Fruits | 240 | 231 | 150 | 167 | 1030 |  | 1037 | 723 | 720 | 692 | 790 |
| Red meat | 21 | 262 | 811 | 1296 | 1129 |  | 2116 | 1394 | 893 | 684 | 1226 |
| Poultry | 0 | 5 | 3 | 3 | 13 |  | 438 | 293 | 183 | 121 | 107 |
| Animal organ meat | 8 | 10 | 9 | 11 | 17 |  | 164 | 120 | 75 | 55 | 43 |
| Processed meat | 57 | 54 | 38 | 20 | 17 |  | 630 | 437 | 313 | 223 | 166 |
| Eggs | 2 | 1 | 1 | 3 | 8 |  | 625 | 415 | 274 | 196 | 144 |
| Fish | 16 | 35 | 23 | 22 | 20 |  | 109 | 67 | 48 | 37 | 33 |
| Other seafood | 76 | 509 | 380 | 318 | 313 |  | 719 | 496 | 331 | 334 | 316 |
| Bean products | 58 | 78 | 62 | 40 | 38 |  | 543 | 406 | 301 | 225 | 201 |
| Nuts | 247 | 493 | 1579 | 1303 | 1120 |  | 480 | 342 | 523 | 1168 | 863 |
| Milk | 23 | 71 | 48 | 41 | 39 |  | 95 | 52 | 35 | 35 | 40 |
| Yoghurt | 40 | 30 | 16 | 19 | 21 |  | 1 | 0 | 3 | 2 | 2 |
| Sweet beverages | 32 | 18 | 12 | 7 | 11 |  | 12 | 11 | 33 | 31 | 17 |
| Cantonese desserts | 2 | 3 | 3 | 4 | 5 |  | 3 | 3 | 2 | 2 | 4 |
| Cantonese soups | 12 | 61 | 35 | 29 | 29 |  | 187 | 120 | 88 | 67 | 56 |
| Puffed food | 5 | 8 | 9 | 8 | 10 |  | 0 | 1 | 0 | 0 | 2 |
| Confectioneries | 34 | 75 | 56 | 52 | 45 |  | 48 | 31 | 24 | 29 | 29 |
| Snack | 3 | 11 | 9 | 12 | 30 |  | 270 | 198 | 130 | 95 | 80 |

The largest values are underlined.

**Table S5** Frequencies of weekly intake of 30 food groups assessed with a self-administered food frequency questionnaire across the six dietary patterns identified among 7352 pregnant Chinese women from the Born in Guangzhou Cohort Study

|  | Dietary patterns-S^*^ | | | | | | | | | | | | | | | | |
| --- | --- | --- | --- | --- | --- | --- | --- | --- | --- | --- | --- | --- | --- | --- | --- | --- | --- |
|  | Rich | |  | Milk-S | |  | Fruits | |  | Meats-S | |  | Moderate | |  | Prudent | |
|  | (n=381) | |  | (n=864) | |  | (n=930) | |  | (n=975) | |  | (n=1735) | |  | (n=2467) | |
| Food groups | Mean | SD |  | Mean | SD |  | Mean | SD |  | Mean | SD |  | Mean | SD |  | Mean | SD |
| Cereals |  |  |  |  |  |  |  |  |  |  |  |  |  |  |  |  |  |
| Rice | 14.0 | 4.3 |  | 12.8 | 3.8 |  | 11.2 | 4.0 |  | 14.0 | 3.7 |  | 12.7 | 3.2 |  | 10.7 | 3.9 |
| Pasta | 6.5 | 4.7 |  | 5.1 | 3.8 |  | 5.3 | 4.1 |  | 5.4 | 3.9 |  | 4.6 | 3.0 |  | 4.0 | 2.7 |
| Noodles | 3.8 | 3.6 |  | 2.0 | 2.4 |  | 3.6 | 4.1 |  | 2.6 | 2.8 |  | 2.3 | 2.6 |  | 2.1 | 2.4 |
| Porridge | 2.5 | 2.6 |  | 1.5 | 1.6 |  | 1.5 | 1.6 |  | 2.0 | 2.3 |  | 1.6 | 1.7 |  | 1.5 | 1.5 |
| Bread | 3.7 | 3.2 |  | 2.1 | 2.5 |  | 1.8 | 2.2 |  | 1.9 | 2.2 |  | 2.0 | 2.0 |  | 1.8 | 1.8 |
| Vegetables |  |  |  |  |  |  |  |  |  |  |  |  |  |  |  |  |  |
| Leafy and cruciferous vegetables | 31.7 | 9.9 |  | 13.2 | 4.5 |  | 11.7 | 4.2 |  | 12.9 | 4.5 |  | 16.7 | 3.8 |  | 7.3 | 2.6 |
| Root vegetables | 7.0 | 4.9 |  | 3.1 | 2.2 |  | 4.4 | 2.8 |  | 3.8 | 2.5 |  | 3.4 | 2.3 |  | 2.5 | 1.7 |
| Melon vegetables | 8.1 | 5.4 |  | 4.1 | 2.7 |  | 5.3 | 3.2 |  | 4.8 | 3.2 |  | 3.8 | 2.5 |  | 3.2 | 2.0 |
| Mushrooms | 2.8 | 2.9 |  | 1.2 | 1.5 |  | 1.9 | 2.0 |  | 1.5 | 1.4 |  | 1.2 | 1.1 |  | 1.0 | 1.0 |
| Sea vegetables | 1.8 | 2.3 |  | 0.8 | 1.1 |  | 1.2 | 1.3 |  | 0.9 | 1.2 |  | 0.7 | 1.0 |  | 0.6 | 0.9 |
| Bean vegetables | 2.7 | 2.1 |  | 1.4 | 1.4 |  | 1.9 | 1.6 |  | 1.8 | 1.6 |  | 1.4 | 1.3 |  | 1.2 | 1.1 |
| Processed vegetables | 0.9 | 1.6 |  | 0.3 | 0.8 |  | 0.7 | 1.3 |  | 0.6 | 1.1 |  | 0.5 | 0.9 |  | 0.4 | 0.8 |
| Fruits | 15.1 | 9.5 |  | 9.7 | 3.8 |  | 16.4 | 6.2 |  | 9.6 | 3.7 |  | 8.1 | 3.0 |  | 7.2 | 2.9 |
| Meats |  |  |  |  |  |  |  |  |  |  |  |  |  |  |  |  |  |
| Red meat | 14.2 | 6.1 |  | 9.3 | 3.8 |  | 8.9 | 3.8 |  | 17.0 | 5.0 |  | 8.2 | 3.2 |  | 6.5 | 3.1 |
| Poultry | 4.8 | 3.5 |  | 2.9 | 2.2 |  | 2.6 | 2.1 |  | 3.3 | 3.5 |  | 2.7 | 2.1 |  | 2.1 | 1.6 |
| Animal organ meat | 2.2 | 3.2 |  | 1.2 | 1.8 |  | 1.1 | 1.5 |  | 1.3 | 1.9 |  | 1.0 | 1.4 |  | 0.9 | 1.3 |
| Processed meat | 0.3 | 1.2 |  | 0.4 | 1.0 |  | 0.3 | 0.9 |  | 0.5 | 1.2 |  | 0.4 | 0.9 |  | 0.3 | 0.9 |
| Eggs | 8.2 | 4.6 |  | 6.9 | 3.4 |  | 6.6 | 2.9 |  | 7.1 | 4.4 |  | 5.5 | 2.5 |  | 4.9 | 2.2 |
| Fish | 5.8 | 4.3 |  | 3.7 | 2.6 |  | 3.6 | 2.6 |  | 3.8 | 2.7 |  | 3.2 | 2.0 |  | 2.6 | 1.8 |
| Other seafood | 1.9 | 2.5 |  | 1.0 | 1.2 |  | 1.3 | 1.5 |  | 1.2 | 1.7 |  | 1.0 | 1.3 |  | 1.0 | 1.2 |
| Bean products | 10.3 | 7.6 |  | 4.2 | 3.3 |  | 8.1 | 5.3 |  | 5.6 | 3.9 |  | 4.3 | 2.9 |  | 4.1 | 2.9 |
| Nuts | 7.4 | 4.9 |  | 5.0 | 3.1 |  | 6.1 | 4.2 |  | 4.6 | 3.0 |  | 4.0 | 2.8 |  | 3.3 | 2.5 |
| Milk | 10.0 | 6.6 |  | 15.5 | 7.3 |  | 6.9 | 3.9 |  | 6.8 | 3.6 |  | 5.4 | 3.0 |  | 5.5 | 3.2 |
| Yoghurt | 3.3 | 3.5 |  | 1.9 | 2.4 |  | 2.8 | 2.6 |  | 2.2 | 2.4 |  | 2.1 | 2.4 |  | 1.9 | 2.1 |
| Sweet beverages | 1.6 | 2.5 |  | 1.5 | 2.9 |  | 1.7 | 2.6 |  | 1.6 | 2.8 |  | 1.4 | 2.5 |  | 1.6 | 2.9 |
| Cantonese desserts | 0.3 | 0.9 |  | 0.4 | 1.1 |  | 0.6 | 1.6 |  | 0.4 | 1.1 |  | 0.3 | 0.8 |  | 0.2 | 0.8 |
| Cantonese soups | 4.4 | 2.3 |  | 4.4 | 2.5 |  | 4.1 | 2.5 |  | 4.8 | 2.5 |  | 4.0 | 2.3 |  | 3.5 | 2.2 |
| Puffed food | 0.2 | 0.7 |  | 0.2 | 0.6 |  | 0.3 | 0.7 |  | 0.2 | 0.7 |  | 0.2 | 0.6 |  | 0.2 | 0.6 |
| Confectioneries | 2.7 | 3.4 |  | 1.6 | 2.4 |  | 2.5 | 3.5 |  | 1.9 | 2.5 |  | 1.7 | 2.3 |  | 1.6 | 2.1 |
| Snack | 5.7 | 5.2 |  | 3.6 | 3.8 |  | 3.1 | 3.1 |  | 3.0 | 2.8 |  | 2.9 | 2.7 |  | 2.6 | 2.4 |
| Total frequencies of food intake | 184 |  |  | 121 |  |  | 127 |  |  | 127 |  |  | 107 |  |  | 86 |  |

^*^These dietary patterns were constructed by using the ‘frequencies of intake of food groups’ as input variables. The highest mean values are underlined.

**Table S6** Characteristics of the participants across the six dietary patterns identified by cluster analysis

|  | Total | Dietary patterns-S | | | | | | | | | | | *P*_value_* |
| --- | --- | --- | --- | --- | --- | --- | --- | --- | --- | --- | --- | --- | --- |
|  |  | Rich |  | Milk-S |  | Fruits |  | Meats-S |  | Moderate |  | Prudent |  |
| Characteristics | (n=7352) | (n=381) |  | (n=864) |  | (n=930) |  | (n=975) |  | (n=1735) |  | (n=2467) |  |
| Age, years, mean ± SD | 29.1 ± 3.3 | 29.2 ± 3.7 |  | 29.1 ± 3.6 |  | 29.1 ± 3.2 |  | 29.0 ± 3.2 |  | 29.2 ± 3.3 |  | 29.0 ± 3.3 | 0.25 |
| Education level, n (%) |  |  |  |  |  |  |  |  |  |  |  |  | <0.01 |
| High school or below | 624 (8.5) | 24 (6.3) |  | 96 (11.1) |  | 69 (7.4) |  | 69 (7.1) |  | 152 (8.8) |  | 214 (8.7) |  |
| Vocational/technical college | 1807 (24.6) | 84 (22.0) |  | 231 (26.7) |  | 191 (20.5) |  | 217 (22.3) |  | 435 (25.1) |  | 649 (26.3) |  |
| Undergraduate | 4031 (54.8) | 225 (59.1) |  | 452 (52.3) |  | 483 (51.9) |  | 557 (57.1) |  | 968 (55.8) |  | 1346 (54.6) |  |
| Postgraduate | 890 (12.1) | 48 (12.6) |  | 85 (9.8) |  | 187 (13.5) |  | 132 (13.5) |  | 180 (10.4) |  | 258 (10.5) |  |
| Monthly income, Yuan, n (%) | | |  |  |  |  |  |  |  |  |  |  | <0.01 |
| ≤1500 | 692 (9.4) | 25 (6.6) |  | 83 (9.6) |  | 83 (8.9) |  | 74 (7.6) |  | 198 (11.4) |  | 229 (9.3) |  |
| 1501-4500 | 2274 (30.9) | 101 (26.5) |  | 330 (38.2) |  | 199 (21.4) |  | 294 (30.2) |  | 547 (31.5) |  | 803 (32.5) |  |
| 4501-9000 | 3062 (41.6) | 178 (46.7) |  | 316 (36.6) |  | 425 (45.7) |  | 427 (43.8) |  | 710 (40.9) |  | 1006 (40.8) |  |
| ≥9001 | 1158 (15.8) | 65 (17.1) |  | 111 (12.8) |  | 194 (16.5) |  | 161 (16.5) |  | 249 (14.4) |  | 378 (15.3) |  |
| Refused to answer | 166 (2.3) | 12 (3.1) |  | 24 (2.8) |  | 29 (3.1) |  | 19 (1.9) |  | 31 (1.8) |  | 51 (2.1) |  |
| Parity, n (%) |  |  |  |  |  |  |  |  |  |  |  |  | <0.01 |
| Primiparous | 6430 (87.5) | 311 (81.6) |  | 792 (91.7) |  | 839 (90.2) |  | 843 (86.5) |  | 1477 (85.1) |  | 2168 (87.9) |  |
| Multiparous | 922 (12.5) | 70 (18.4) |  | 72 (8.3) |  | 91 (9.8) |  | 132 (13.5) |  | 258 (14.9) |  | 299 (12.1) |  |
| Passive smoking during pregnancy, n (%) | 2222 (30.2) | 96 (25.2) |  | 252 (29.2) |  | 250 (26.9) |  | 312 (32.0) |  | 529 (30.5) |  | 783 (31.7) | <0.01 |
| Supplementation with folic acid, n (%) | | |  |  |  |  |  |  |  |  |  |  | 0.11 |
| No | 603 (8.2) | 33 (8.7) |  | 63 (7.3) |  | 57 (6.1) |  | 75 (7.7) |  | 156 (9.0) |  | 219 (8.9) |  |
| Started during pregnancy | 3501 (47.6) | 185 (48.6) |  | 416 (48.1) |  | 378 (40.6) |  | 441 (45.2) |  | 856 (49.3) |  | 1225 (49.7) |  |
| Started pre- conception | 3248 (44.2) | 163 (42.8) |  | 385 (44.6) |  | 495 (53.2) |  | 459 (47.1) |  | 723 (41.7) |  | 1023 (41.5) |  |
| Pre-pregnancy BMI, kg/m^2^, n (%) | | |  |  |  |  |  |  |  |  |  |  | 0.03 |
| <18.5 | 1803 (24.5) | 98 (25.7) |  | 237 (27.4) |  | 228 (24.5) |  | 233 (23.9) |  | 431 (24.8) |  | 576 (23.3) |  |
| 18.5-23.9 | 4608 (62.7) | 241 (63.3) |  | 502 (58.1) |  | 611 (65.7) |  | 624 (64.0) |  | 1055 (60.8) |  | 1575 (63.8) |  |
| ≥24 | 847 (11.5) | 35 (9.2) |  | 112 (13.0) |  | 82 (8.8) |  | 104 (10.7) |  | 228 (13.1) |  | 286 (11.6) |  |
| Missing | 94 (1.3) | 7 (1.8) |  | 13 (1.5) |  | 9 (1.4) |  | 14 (1.4) |  | 21 (1.2) |  | 30 (1.2) |  |
| Previous preterm delivery, n (%) | 53 (0.7) | 4 (1.0) |  | 8 (0.9) |  | 8 (0.9) |  | 7 (0.7) |  | 16 (0.9) |  | 10 (0.4) | 0.33 |

*ANOVA and Chi square tests were used to test differences between the patterns.

**Table S7** Associations between dietary patterns and preterm delivery

|  | Dietary patterns-S | | | | | | | | | | |
| --- | --- | --- | --- | --- | --- | --- | --- | --- | --- | --- | --- |
|  | Rich |  | Milk-S |  | Fruits |  | Meats-S |  | Moderate |  | Prudent |
| Preterm delivery | (n=381) |  | (n=864) |  | (n=930) |  | (n=975) |  | (n=1735) |  | (n=2467) |
| Overall preterm delivery (n, %) | 13 (3.4) |  | 52 (6.0) |  | 47 (5.1) |  | 37 (3.8) |  | 84 (4.8) |  | 118 (4.8) |
| Crude OR (95% CI) | 0.69 (0.39-1.22) |  | 1.33 (0.98-1.80) |  | 1.07 (0.78-1.47) |  | 0.76 (0.54-1.09) |  | 1.02 (0.79-1.31) |  | 1.00 (0.80-1.26) |
| Adjusted OR (95% CI) ^1^ | 0.68 (0.39-1.20) |  | 1.30 (0.96-1.77) |  | 1.10 (0.80-1.51) |  | 0.78 (0.55-1.11) |  | 0.99 (0.77-1.28) |  | 1.02 (0.81-1.28) |
| Spontaneous preterm delivery (n, %) | 6 (1.6) |  | 42 (4.9) |  | 36 (3.9) |  | 26 (2.7) |  | 64 (3.7) |  | 88 (3.6) |
| Crude OR (95% CI) | 0.42 (0.19-0.96) ^*^ |  | 1.46 (1.04-2.04) ^*^ |  | 1.10 (0.77-1.58) |  | 0.70 (0.47-1.07) |  | 1.05 (0.79-1.40) |  | 1.00 (0.77-1.30) |
| Adjusted OR (95% CI) ^1^ | 0.41 (0.18-0.93) ^*^ |  | 1.44 (1.02-2.02) ^*^ |  | 1.13 (0.78-1.62) |  | 0.73 (0.48-1.10) |  | 1.03 (0.77-1.38) |  | 1.01 (0.77-1.31) |
| Iatrogenic preterm delivery (n, %) | 6 (1.6) |  | 8 (1.0) |  | 7 (0.8) |  | 5 (0.5) |  | 16 (1.0) |  | 20 (0.8) |
| Crude OR (95% CI) | 1.93 (0.83-4.51) |  | 1.13 (0.54-2.38) |  | 0.88 (0.40-1.94) |  | 0.57 (0.23-1.42) |  | 1.13 (0.64-2.00) |  | 0.94 (0.55-1.61) |
| Adjusted OR (95% CI) ^1, 2^ | 2.08 (0.83-4.42) |  | 1.09 (0.48-2.14) |  | 0.93 (0.40-1.90) |  | 0.63 (0.23-1.40) |  | 1.14 (0.63-1.98) |  | 0.98 (0.57-1.65) |
| Late preterm delivery (n, %) | 8 (2.1) |  | 41 (4.8) |  | 43 (4.6) |  | 29 (3.0) |  | 70 (4.1) |  | 103 (4.2) |
| Crude OR (95% CI) | 0.50 (0.25-1.03) |  | 1.24 (0.88-1.73) |  | 1.19 (0.85-1.65) |  | 0.71 (0.48-1.04) |  | 1.01 (0.77-1.33) |  | 1.07 (0.84-1.36) |
| Adjusted OR (95% CI) ^1^ | 0.50 (0.25-1.02) |  | 1.23 (0.87-1.72) |  | 1.19 (0.85-1.67) |  | 0.72 (0.49-1.07) |  | 0.99 (0.75-1.30) |  | 1.08 (0.85-1.39) |
| Moderately or early preterm delivery (n, %) | 5 (1.3) |  | 11 (1.3) |  | 4 (0.5) |  | 8 (0.8) |  | 14 (0.8) |  | 15 (0.6) |
| Crude OR (95% CI) | 1.73 (0.69-4.37) |  | 1.82 (0.94-3.53) |  | 0.52 (0.19-1.45) |  | 1.06 (0.50-2.24) |  | 1.06 (0.58-1.93) |  | 0.71 (0.39-1.28) |
| Adjusted OR (95% CI) ^1, 2^ | 1.84 (0.67-4.11) |  | 1.76 (0.87-3.27) |  | 0.67 (0.22-1.59) |  | 1.16 (0.52-2.29) |  | 1.01 (0.54-1.80) |  | 0.72 (0.39-1.27) |

^1^ Adjusted for maternal age, education level, monthly income, parity, passive smoking during pregnancy, supplementation with folic acid, pre-pregnancy BMI, and previous preterm delivery.

^2^ The firth’s correction was applied to improve the accuracy of the logit coefficients.

^*^ *P* _value_<0.05
